# Buffalopox Disease in Livestock and Milkers, India

**DOI:** 10.3201/eid2707.202111

**Published:** 2021-07

**Authors:** Parimal Roy, Andrew Chandramohan

**Affiliations:** Tamil Nadu Veterinary and Animal Sciences University, Chennai, India

**Keywords:** buffalopox, viruses, zoonoses, India

## Abstract

Buffalopox outbreaks caused by vaccinia virus were observed in villages of Tamil Nadu, India, among lactating buffaloes and cows. Milkers also had lesions on their fingers. Because vaccinia virus is known to have extended its host range in Brazil, we recommend continuous surveillance to understand cross-species transmission and to curtail disease effects.

In India, sporadic outbreaks of buffalopox, which can be caused by vaccinia virus (VACV), have been reported among cattle and buffaloes (*1*–*3*) and also in humans (*3*). We describe an outbreak affecting 120 lactating buffaloes and 40 lactating cows in Kannivadi, Navapatti, Alathuranpatti, Maniakaranpatti, Muthukumaranpatti, S.Pudur, and E.Chittor, Dindigul district; and in Krishnarayapuram, Karur district, in Tamil Nadu, India in 2004. Pock lesions (0.5–1 cm diameter) were seen over the bodies of lactating buffaloes but restricted to only the udder and teats of lactating cows (Figure, panel A). Buffalopox did not cause death in the animals we reviewed; it affected more buffaloes (30%–50%) than cows (20%–30%). Suckling calves developed pock lesions on the forehead, lips, and mouth. Three milkers who worked with the affected animals experienced multiple pock lesions (1 cm diameter) on the fingers, interdigital webs, wrist and forearm (Figure, panel B) and generalized effects including fever (100°F) and enlargement of axillary lymph nodes. 

**Figure:**
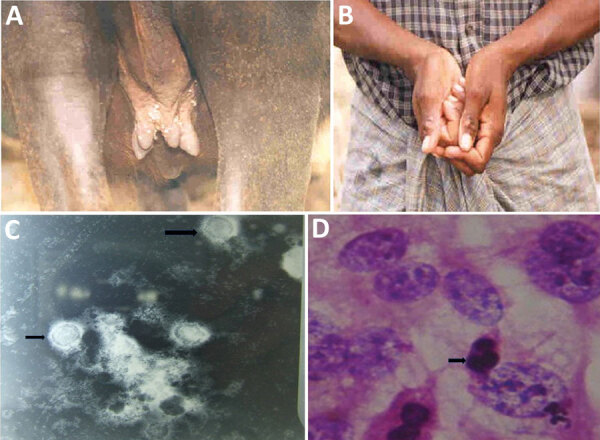
Buffalopox outbreak caused by vaccinia virus, Tamil Nadu, India. A) Buffalopox lesions on the udder and teats and over the body of a buffalo. B) Suspected buffalopox in a milker; lesions are visible on the fingers and forearms. C) Electron micrograph of vaccinia virus (arrows; magnification ×20,000). D) Hematoxylin and eosin stain (magnification ×1,000) shows cellular rounding and cell fusion and intracytoplasmic inclusion bodies (arrow). PCR revealed vaccinia virus infection.

To investigate the causative agent, we used existing clinical samples. Scab samples were collected randomly from 20 affected animals (both buffaloes and cows). We examined scab suspensions under transmission electron microscope (EM) at 80 KV and inoculated the suspension in BHK21 cell line for virus isolation. We examined scab homogenates and cell culture fluid by PCR for differential diagnosis of cowpox virus and VACV infection (*4*). EM revealed typical brick-shaped pox virus particles of ≈290 × 270 nm with irregularly arranged superficial filaments formed by tubules (Figure, panel C). After 2 blind passages, we noticed in BHK21 cell lines cytopathogenic effects such as cellular rounding, cellular fusion, and intracytoplasmic inclusion bodies (Figure, panel D) after 48–60 hours of infection; PCR analysis revealed the causative agent to be VACV. 

During the global eradication of smallpox, strains of VACV were used as vaccine. VACV infection sometimes transmitted from the vasicular lesion of vaccinae to domestic animals, usually cattle; in turn, infected animals transmitted VACV to susceptible humans (*5*). Several outbreaks in cattle and humans that were thought to be cowpox were in fact caused by VACV (*1*,*6*,*7*). The infected animals were treated with parenteral injection of antimicrobial drugs for 1 week to control secondary bacterial infection and an antiinflammatory drug for 3 days to reduce pain and inflammation. Animal workers were also advised to clean the animals’ lesions with 1% potassium permanganate solution followed by tropical application over the pock lesions with indigenous product of neem leaf extract and turmeric powder suspended in glycerin. Individual animals recovered in ≈1 month. Similarly affected humans were diagnosed at primary healthcare centers and treated with oral antimicrobial drugs and analgesics for 1 week, which reduced pain and pustules. Healing was complete in 3 weeks’ time. 

The outbreaks resulted in financial loss to the farmers because of mastitis and loss of milk production. The outbreaks also created public health concern because of human infection. The source of infection could not be identified; it is possible that VACV could be lurking in rodents, as reported earlier (*8*), and causing sporadic outbreaks. More recently, Lima et al. reported that host range of VACV in Brazil has extended over the period 1960–2018; VACV has been detected in rodents, primates, and several species of domesticated animals as well as humans (*9*). Thus, continuous surveillance and the study of genetic diversity of VACV and its pathogenic attributes will be helpful to understand its founder effects and host diversity. Awareness among the stakeholders and steps taken for biosecurity will reduce the transmission of disease in animals and humans.
